# Metastasis of renal cell carcinoma to the urethra: a rare scenario

**DOI:** 10.1590/S1677-5538.IBJU.2020.0719

**Published:** 2020-12-20

**Authors:** Sofia Frade Santos, Pedro Oliveira Santos, Delfim Doutel, José Venâncio

**Affiliations:** 1 Instituto Português de Oncologia de Lisboa Francisco Gentil Lisboa Portugal Instituto Português de Oncologia de Lisboa Francisco Gentil – IPOLFG, Lisboa, Portugal

## INTRODUCTION

Urethral metastasis from renal cell carcinoma is extremely rare, especially in the absence of other metastatic sites.

We present a case of a urethral solitary lesion corresponding to metastasis of a previously diagnosed clear cell renal cell carcinoma.

## CASES PRESENTATION

**Figure 1:**
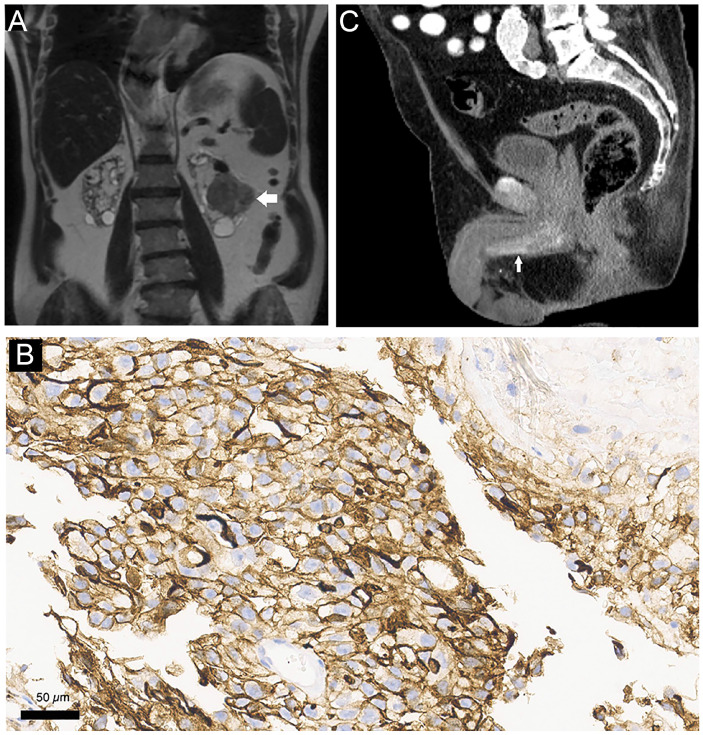
(A-C) – MRI (A: coronal T2WI) demonstrates a solid renal lesion (horizontal arrow in A) in the left kidney, which was proved to represent renal cell carcinoma. The lesion has irregular contours and heterogeneous signal intensity in T2WI. Polycystic kidney disease is also evident with multiple bilateral renal cysts. Pathological analysis of an endoscopically resected urethral lesion confirmed metastasis from RCC. The tumor cells are CD10 positive (B: CD10, x400). A CT performed two months after resection (C: sagittal image after intravenous contrast injection, arterial phase) shows early enhancement (vertical arrow in C) Along the penile bulb and proximal body of the penis, raising suspicion for regrowth of the metastatic lesion.

We report the case of a 67-year-old man with an acute episode of gross hematuria with no reported trauma, fever, dysuria, or flank pain. Six months before admission the patient underwent radical left nephrectomy for clear cell renal cell carcinoma (RCC) (Figure-1A). On histological analysis, the tumor invaded the perinephric fat without signs of vascular invasion nor renal sinus invasion. The surgical margins were free of tumor.

The patient's past medical history was also remarkable for chronic renal failure caused by polycystic kidney disease.

A cystourethroscopy revealed a polypoid lesion in the bulbous urethra, along an approximate length of 3cm. It was resected endoscopically and pathological analysis (Figure-1B) confirmed infiltration by carcinoma morphologically similar to the previously resected renal tumor.

He was readmitted two months after endoscopic resection with recurrent hematuria and, subsequently, an acute urinary retention episode. A urinary catheter was placed after some technical difficulties and a hemorrhage.

A computed tomography (CT) (Figure 1-C) showed early enhancement after contrast injection along the penile bulb and proximal body of the penis.

**Figure 2:**
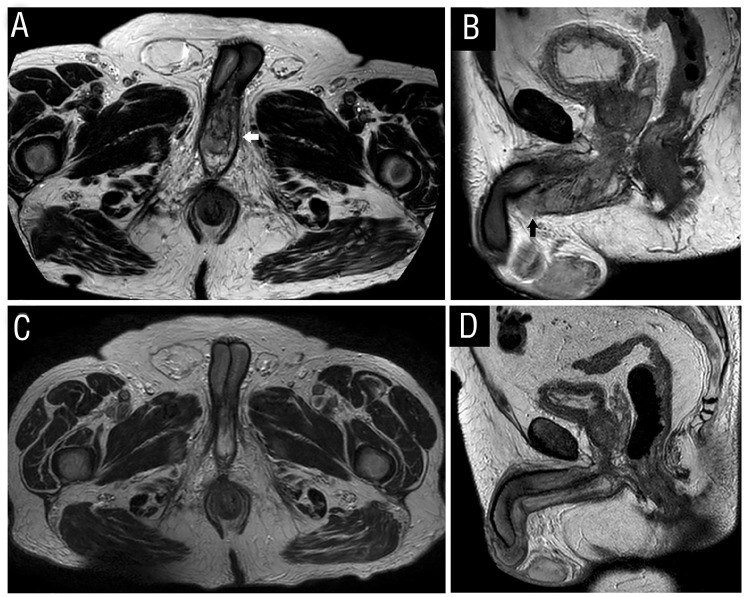
(A-D) – MRI of the urethral metastasis (T2WI). Axial (A) and sagittal (B) images show an area of marked T2 heterogeneity involving the bulb (white arrow in A) and proximal body of the penis (black arrow in B) with a gross cylindrical shape. This lesion has an estimated total extension of 10 cm. It is presumably centred in the urethra (penile, bulbous and membranous) with loss of the normal anatomic planes between the urethra and the surrounding corpus spongiosum. The described lesion corresponds to an area with heterogeneous contrast enhancement after gadolinium injection and restricted diffusion (not shown). Followup MRI of the pelvis after three therapeutic cycles with sunitinib revealed a reduction of the lesion's extension and less heterogeneity, with a better definition of the anatomical planes (C and D). There was no significant restriction to diffusion on DWI (not shown).

Magnetic resonance imaging (MRI) demonstrated an area of marked T2 heterogeneity (Figures 2A and B) involving the bulb and proximal body of the penis with heterogeneous contrast enhancement after gadolinium injection and restrictive on diffusion-weighted imaging (DWI). There was a loss of the regular anatomic planes between the urethra and the surrounding corpus spongiosum.

These findings were suspicious for regrowth of the metastatic lesion. There were no other suspected metastases.

The patient was considered not eligible for surgery, therefore therapy with sunitinib was initiated. This treatment was temporarily interrupted due to toxicity manifested as asthenia, thrombocytopenia, and cardiotoxicity.

After three therapeutic cycles, there was no recurrence of haematuria nor the development of significant lower urinary tract symptoms. The follow-up MRI (Figures 2C and D) showed a reduction in the lesion's extension. The morphological MRI sequences, namely T2-weighted images (T2WI), also confirmed less heterogeneity with a better definition of the anatomical planes. Unequivocal restricted diffusion was no longer evident.

## DISCUSSION

Renal cell carcinoma (RCC) is the most common primary renal malignancy, with clear cell RCC being the most frequent histological subtype (1).

The lung, bone, liver, and adrenal gland are common metastatic sites of RCC (1,2). The urethra is one of the infrequent metastatic sites. To our knowledge, there are only eight cases reported in the literature (3–10), mostly in male patients. Only some represent solitary metastases. In our case, the first metastatic site was the bulbous urethra.

Urethral metastases from other primary sites are also rare, although they are more common than primary urethral tumors (11). The length of the urethra is much larger in men than in women, and that fact may explain the higher rates of urethral metastases in male patients (10). MRI is considered the imaging modality of choice in the assessment of secondary urethral tumors to depict the location, size, and local extension due to proper soft-tissue resolution (11). Extensive secondary tumoral involvement of the urethra presents as a solid nodular or cylindrical mass with heterogeneous contrast enhancement (11), as in our case.

During follow-up imaging (CT and MRI), the recurrence of the histologically proved metastatic lesion was suspected. Clinical and imaging data showed a favourable response to sunitinib, a tyrosine kinase inhibitor used in the treatment of metastatic RCC (12–14).

The lesion reported on MRI (Figure-2A-B) caused the loss of the anatomical planes between the urethra and corpus spongiosum. Probably, it is explained by direct extension of the known urethral metastasis to the surrounding corpus spongiosum. The penis is a rare site of metastases and usually affects the corpora cavernosa, while corpus spongiosum invasion is rare (15,16). Most penile metastatic lesions are associated with disseminated metastatic disease and carry an overall poor prognosis (15,17). Urethral and penile metastases may be explained by hematogenous, instrumental and, retrograde lymphatic spread. Dissemination through the urinary excretory system or even direct extension should also be considered (10,11). Spread of tumor cells through the distal urinary excretory system may be favored by the location of a primary tumor in the kidney (close to the renal collecting system) (10).

Infrequent sites of metastatic disease may delay its detection (1), as illustrated by our case. In patients with a known primary malignancy, a metastatic deposit, although rare, should be considered as a cause for the development of lower urinary tract symptoms.

## abbreviations

RCC: renal cell carcinoma;
CT: computed tomography;
MRI: magnetic resonance imaging;
DWI: diffusion-weighted imaging;
T2WI: T2-weighted images

## References

[1] 1. Diaz de Leon A, Pirasteh A, Costa DN, Kapur P, Hammers H, Brugarolas J, et at. Current Challenges in Diagnosis and Assessment of the Response of Locally Advanced and Metastatic Renal Cell Carcinoma. Radiographics. 2019; 39:998-1016. 10.1148/rg.2019180178 PMC6677287 31199711

[2] 2. Griffin N, Gore ME, Sohaib SA. Imaging in metastatic renal cell carcinoma. AJR Am J Roentgenol. 2007; 189:360-70. 10.2214/AJR.07.2077 17646462

[3] 3. Goldberg MG, Plaine L. Solitary metastasis of renal cell carcinoma to urethra. Urology. 1990; 35:351-3. 10.1016/0090-4295(90)80164-i 2321330

[4] 4. Fukata S, Inoue K, Moriki T, Shuin T. A solitary metastasis of renal cell carcinoma to the urethra. J Urol. 2000; 163:1245-6. 10737510

[5] 5. Senzaki H, Okamura T, Tatsura H, Watase H. Urethral metastasis from renal cell carcinoma. Int J Urol. 2003; 10:661-3. 10.1046/j.1442-2042.2003.00717.x 14633070

[6] 6. Cheng CW, Wong WS, Chan LW, Lai FM. A rare cause of acute urinary retention: urethral metastasis from renal cell carcinoma. Int Urol Nephrol. 2004; 36:145-7. 10.1023/b:urol.0000034667.78108.b9 15368681

[7] 7. Bailie J, Wood E, Connolly D, O’Rourke D. Urethral metastasis from renal cell carcinoma: An unusual cause of visible painless haematuria. J Clin Urol.2012, 6:188-90.

[8] 8. Kravvas G, Varnavas M, Aldujaily S. A unique presentation of an undiagnosed renal cell carcinoma. Case Rep Urol. 2014;2014:840163840163. 10.1155/2014/840163 PMC4241245 25431735

[9] 9. Pirola GM, Martorana E, Fidanza FA, Bonetti LR, Puliatti S, Bonora A, et at. Rare metastatic sites of renal cell carcinoma: urethra and spermatic cord. Urologia. 2016; 83:214-7. 10.5301/uro.5000200 27739563

[10] 10. Gawlik-Jakubczak T, Matuszewski M, Biernat W. The Metastasis of Renal Cell Carcinoma to the Urethra and Local Tumor Recurrence. Urol Int. 2020; 104:327-9. 10.1159/000501699 31694043

[11] 11. Karaosmanoglu AD, Onur MR, Karcaaltincaba M, Akata D, Ozmen MN. Secondary Tumors of the Urinary System: An Imaging Conundrum. Korean J Radiol. 2018; 19:742-51. 10.3348/kjr.2018.19.4.742 PMC6005933 29962880

[12] 12. Rizzo M, Porta C. Sunitinib in the treatment of renal cell carcinoma: an update on recent evidence. Ther Adv Urol. 2017; 9:195-207. 10.1177/1756287217713902 PMC5896861 29662544

[13] 13. Toussi A, Agarwal D, Leibovich B, Potretzke A. Percutaneous resection of metastatic renal cell carcinoma. Int Braz J Urol. 2019; 45:640640. 10.1590/S1677-5538.IBJU.2018.0490 PMC6786114 30620159

[14] 14. Wang B, Gu W, Wan F, Shi G, Ye D. Prognostic significance of the dynamic changes of systemic inflammatory response in metastatic renal cell carcinoma. Int Braz J Urol. 2019; 45:89-99. 10.1590/S1677-5538.IBJU.2017.0500 PMC6442152 29570259

[15] 15. Mearini L, Colella R, Zucchi A, Nunzi E, Porrozzi C, Porena M. A review of penile metastasis. Oncol Rev. 2012; 6:e10. 10.4081/oncol.2012.e10 PMC4419641 25992200

[16] 16. Nezu FM, Dhir R, Logan TF, Lavelle J, Becich MJ, Chancellor MB. Malignant priapism as the initial clinical manifestation of metastatic renal cell carcinoma with invasion of both corpora cavernosum and spongiosum. Int J Impot Res. 1998;10:101101. 10.1038/sj.ijir.3900342 9647945

[17] 17. Romero Selas E, Lamas Meilán C, Barbagelata López A, Ponce Díaz-Reixa JL, Fernández Rosado E, Alvarez Castelo L, et al. Metastasis de carcinoma renal en cuerpo cavernoso, a propósito de un caso y revisión de la literatura [Metastasis of a renal cell carcinoma in the corpora cavernosum of the penis. Case report and bibliographic review]. Arch Esp Urol. 2006;59:530-2. Spanish. 10.4321/s0004-06142006000500012 16903557

